# Acceleration of the Glycolytic Flux by Steroid Receptor Coactivator-2 Is Essential for Endometrial Decidualization

**DOI:** 10.1371/journal.pgen.1003900

**Published:** 2013-10-24

**Authors:** Ramakrishna Kommagani, Maria M. Szwarc, Ertug Kovanci, William E. Gibbons, Nagireddy Putluri, Suman Maity, Chad J. Creighton, Arun Sreekumar, Francesco J. DeMayo, John P. Lydon, Bert W. O'Malley

**Affiliations:** 1 Department of Molecular and Cellular Biology, Baylor College of Medicine, Houston, Texas, United States of America; 2 Department of Obstetrics and Gynecology, Baylor College of Medicine, Houston, Texas, United States of America; 3 Alkek Center for Molecular Discovery, Baylor College of Medicine, Houston, Texas, United States of America; 4 Dan L. Duncan Cancer Center, Baylor College of Medicine, Houston, Texas, United States of America; Massachusetts General Hospital/Harvard Medical School, United States of America

## Abstract

Early embryo miscarriage is linked to inadequate endometrial decidualization, a cellular transformation process that enables deep blastocyst invasion into the maternal compartment. Although much of the cellular events that underpin endometrial stromal cell (ESC) decidualization are well recognized, the individual gene(s) and molecular pathways that drive the initiation and progression of this process remain elusive. Using a genetic mouse model and a primary human ESC culture model, we demonstrate that steroid receptor coactivator-2 (SRC-2) is indispensable for rapid steroid hormone-dependent proliferation of ESCs, a critical cell-division step which precedes ESC terminal differentiation into decidual cells. We reveal that SRC-2 is required for increasing the glycolytic flux in human ESCs, which enables rapid proliferation to occur during the early stages of the decidualization program. Specifically, SRC-2 increases the glycolytic flux through induction of 6-phosphofructo-2-kinase/fructose-2, 6-bisphosphatase 3 (PFKFB3), a major rate-limiting glycolytic enzyme. Similarly, acute treatment of mice with a small molecule inhibitor of PFKFB3 significantly suppressed the ability of these animals to exhibit an endometrial decidual response. Together, these data strongly support a conserved mechanism of action by which SRC-2 accelerates the glycolytic flux through PFKFB3 induction to provide the necessary bioenergy and biomass to meet the demands of a high proliferation rate observed in ESCs prior to their differentiation into decidual cells. Because deregulation of endometrial SRC-2 expression has been associated with common gynecological disorders of reproductive-age women, this signaling pathway, involving SRC-2 and PFKFB3, promises to offer new clinical approaches in the diagnosis and/or treatment of a non-receptive uterus in patients presenting idiopathic infertility, recurrent early pregnancy loss, or increased time to pregnancy.

## Author Summary

Failure of an embryo to correctly implant into the endometrium is a common cause of pregnancy failure or early embryo miscarriage. Although advances in our understanding of oocyte and embryo development have significantly increased pregnancy success rates, these rates remain unacceptably low due in part to an endometrium that is unreceptive to embryo implantation. Using experimental mouse genetics and a primary human cell culture model, we show here that the development of a receptive endometrium requires steroid receptor coactivator-2, a factor which modulates the response of an endometrial cell to the pregnancy hormone, progesterone. Specifically, we show that SRC-2 increases progesterone-dependent glycolysis in the endometrial cell to provide energy and biomolecules for the next round of cell division. For an endometrium to be receptive to embryo implantation, specific endometrial cells (termed stromal cells) need to divide and numerically increase just prior to development of the receptive state. Therefore, SRC-2 is critical for the metabolic reprogramming of the endometrium to a receptive state, which provides the pretext for considering this factor and its metabolic targets in the design of future clinical approaches to diagnose and therapeutically treat those women at a high risk for early pregnancy loss.

## Introduction

Progression of embryo implantation into a receptive endometrium relies on endometrial stromal cells (ESCs) undergoing decidualization, a critical cellular transformation process which determines the depth of embryo invasion and placentation [1]–[3]. Therefore, inadequate decidualization of ESCs can lead to embryo miscarriage and early pregnancy loss irrespective of whether the development of the blastocyst is normal. Prior to ESC decidualization, the endometrium must first transition from a “pre-receptive” to a “receptive” state within a restricted time period (the window of receptivity) during which the endometrial epithelium is transiently responsive to embryo attachment and invasion [4]. Accordingly, successful embryo implantation can only occur following precise synchronization between the emergence of an “activated” blastocyst and the differentiation of the endometrium to a “receptive” state. From a clinical perspective, the inability to diagnose or therapeutically treat a uterus that is incapable of developing a receptive state is considered one of the remaining obstacles to substantially improving the efficacy of assisted reproductive technologies (ARTs) which rely on embryo-transfer into a receptive uterus [5], [6].

In the human and mouse endometrium, the onset of the receptive state is controlled by the coordinated actions of ovarian derived estradiol-17β (E2) and progesterone (P4) [4]. Although the initial cellular events of embryo implantation in the human are interstitial as opposed to eccentric in the mouse [1], the fundamental developmental steps that lead to the establishment of the receptive uterus are common to both species, suggesting that many of the biochemical and molecular mechanisms underlying E2 and P4 control of these developmental steps also are conserved. During the proliferative (or follicular) phase of the human menstrual cycle or estrous cycle in the mouse, rising preovulatory E2 levels induce proliferation of the luminal and glandular epithelia of the endometrium. Following ovulation, however, E2-induced epithelial proliferation is suppressed by P4 derived from newly formed corpora lutea, resulting in a shift from epithelial proliferation to differentiation. Within this early secretory (or luteal phase) of the cycle, the anti-proliferative action of P4 in the epithelium is paralleled by rapid P4 induced proliferation of subepithelial stromal fibroblasts and their subsequent differentiation into decidual cells [7]. Unlike their fibroblast progenitors, decidual cells are large polygonal epithelioid cells which in many cases are polyploid. In the case of the day 4 pregnant mouse (day 1 = detection of vaginal plug), ESC proliferation is further enhanced by nidatory E2 to elicit an endometrial receptive state [4]. As a consequence of embryo attachment (or an artificial deciduogenic stimulus), proliferating ESCs differentiate into polyploid multinucleated decidual cells [8]. The resulting decidua expands mesometrially to ultimately contribute to the formation of the chorioallantoic placenta (hemochorial placentation also occurs in the human [1]). Importantly, many of the above endometrial cellular events that lead to the establishment of the materno-fetal interface in the human and mouse can be recapitulated in ovariectomized mice treated with E2 and P4 [9].

Although the key cellular events that underpin the development of endometrial receptivity by E2 and P4 are known, the biochemical and molecular mechanisms by which these steroid hormones choreograph these intracellular changes are unclear. To address this issue, we previously demonstrated that of the three members of the p160/steroid receptor coactivator (p160/SRC) family of coregulators [10], endometrial SRC-2 is indispensable for successful embryo implantation in the mouse [11], [12]. Significantly, these initial mouse findings provided much needed experimental support for conclusions drawn from clinical studies that deregulation of endometrial SRC-2 expression levels is linked to the infertility diagnosis often made for a subset of patients presenting gynecological disorders such as polycystic ovary syndrome (PCOS) [13].

Despite growing support for an important role for SRC-2 in endometrial function and disease processes [12]–[16], the signaling mechanisms by which this endometrial coregulator exerts its effects have remained elusive. Therefore, using primary human ESCs (hESCs) in culture and a conditional knockout mouse model, we report here that SRC-2 is required for early P4-driven ESC proliferation which is essential for the rapid numerical increase of both human and mouse decidual cells during the decidualization progression program. In keeping with SRC-2's pleiotropic role in glucose metabolism [17]–[19], we reveal that SRC-2 enables P4-driven ESC proliferation through accelerating the glycolytic flux in order to provide the necessary bioenergy and biomolecules (*i.e.* amino acids, fatty acids, and nucleotides) to meet the demands of rapid cellular proliferation during this early stage of the decidualization program. Therefore, our new findings support the conclusion that SRC-2 represents a critical “coregulator accelerant” of the glycolytic flux which is required to rapidly expand the ESC population prior to terminal differentiation to decidual cells. Accordingly, endometrial SRC-2 and its glycolytic targets may represent novel clinical avenues through which to precisely time and/or therapeutically extend the window of receptivity in those women at high risk for early pregnancy loss.

## Results

### Endometrial SRC-2 is essential for estrogen and progesterone-driven ESC proliferation which is required for murine uterine receptivity

**Figure 1:**
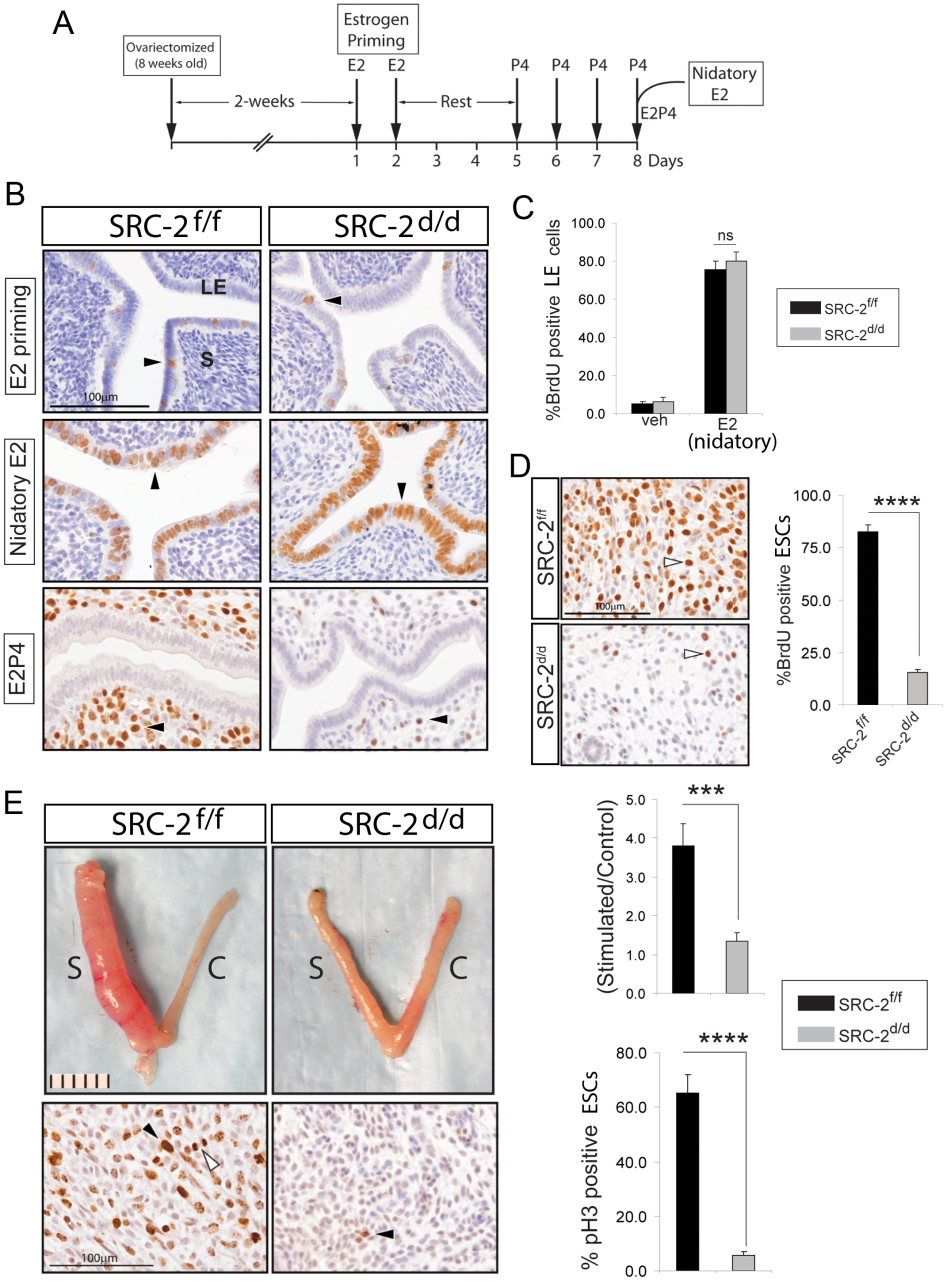
Endometrial receptivity requires SRC-2 dependent ESC proliferation in the mouse. (A) Time-line for hormone induction of uterine receptivity in the ovariectomized mouse (Methods and [20]). (B) Immunohistochemical detection of BrdU incorporation in uteri derived from SRC-2^f/f^ and SRC-2^d/d^ mice following E2 priming alone, E2 priming followed by nidatory E2 alone, or E2 priming followed by E2P4. Arrowheads indicate cells immunopositive for BrdU. Scale bar applies to all panels; “LE” and “S” denote luminal epithelium and stroma respectively. (C) Percentage of luminal epithelial cells scoring positive for BrdU immunopositivity in uteri derived from SRC-2^f/f^ and SRC-2^d/d^ mice treated with nidatory E2 alone. (D) Top and bottom panels show immunohistochemical detection of BrdU incorporation in ESCs derived from uteri of SRC-2^f/f^ and SRC-2^d/d^ mice respectively following E2P4 treatment. Proliferating ESCs are indicated by arrowheads. Histogram on right displays percentage of BrdU positive ESCs in SRC-2^f/f^ and SRC-2^d/d^ uteri following E2P4 treatment. Results represent means ± SE; n = 5mice/group (****P<0.0001). (E) Top panels show gross morphology of uteri from hormone treated SRC-2^f/f^ and SRC-2^d/d^ mice two days following receipt of a deciduogenic stimulus (see Methods and Figure S1 for further details). Scale bar applies to both panels; “S” and “C” denote stimulated and control horn respectively. Top right histogram displays wet-weight ratios of stimulated over control uterine horns of SRC-2^f/f^ and SRC-2^d/d^ mice. Bottom panels show immunohistochemical detection of phospho-histone H3 (pH3) in the stimulated horns of SRC-2^f/f^ (left panel) and SRC-2^d/d^ (right panel) mice. Black arrowheads indicate ESCs undergoing proliferation; mitotic figure is indicated by a white arrowhead. Scale bar applies to both panels. Lower right histogram shows the percentage of ESCs undergoing proliferation in the stimulated horn of the SRC-2^f/f^ and SRC-2^d/d^ mice two days following receipt of the deciduogenic stimulus. Percentage of pH3 positive cells was calculated by counting pH3 positive cells from a total of 200 subepithelial ESCs in three separate fields. Results represent mean± SE; n = 5 mice/group. *P<0.05; **P<0.01; ***P<0.01 and ****P<0.0001.

Our previous murine endometrial studies demonstrated that SRC-2 is critical for the process of ESC decidualization [12]; however, the cellular and molecular mechanism(s) which rely on SRC-2 to advance this decidual progression program by E2 and P4 is unclear. Using an established hormone-induced uterine receptivity model in which the sequential and co-stimulatory actions of E2 and P4 elicit a receptive uterus in an ovariectomized mouse (Figure 1A and [20]), we asked whether abrogation of SRC-2 expression in the endometrium of the SRC-2^d/d^ mouse would compromise the responsiveness of this tissue to E2 and/or P4 exposure. Immunostaining for BrdU incorporation clearly revealed that abrogation of SRC-2 in the SRC-2^d/d^ mouse uterus is not required for endometrial epithelial proliferation in response to E2 priming or nidatory E2 exposure (Figure 1B and C). However, a significant decrease in the number of subepithelial BrdU positive ESCs was observed in uterus of the SRC-2^d/d^ mouse as compared to its SRC-2^f/f^ sibling control when treated with E2P4 (Figure 1B and D). In an estrogenized endometrium, these data provide support for a critical role for SRC-2 in P4-dependent ESC proliferation, an essential cellular event for developing a receptive uterus responsive to a deciduogenic stimulus. Using an artificial decidual assay (Figure S1A and [9]), we confirmed that the marked decrease in the number of proliferating ESCs resulting from the absence of SRC-2 is sufficient to block the responsiveness of the uterus to a deciduogenic stimulus (Figure 1E and Figure S1). As expected, P4 responsive ESC genes induced during early decidualization (*i.e. Wnt-4*, *Bmp-2*, and *Hand-2* [4], [21]–[24]) are strikingly attenuated in the absence of SRC-2 function (Figure S1B). Additionally, expression of cell cycle regulatory genes *Ccnd3, Cdc25c and Cdkn1a* were significantly altered with the absence of SRC-2 function (Figure S6). Together, these results provide strong support for a pivotal role for uterine SRC-2 in P4-dependent ESC proliferation that initiates with the development of the receptive uterus and is required to advance a cellular transformation program which leads to terminal differentiation and complete decidualization of the ESC compartment within the murine endometrium.

### Early requirement for SRC-2 during hESC decidualization

**Figure 2:**
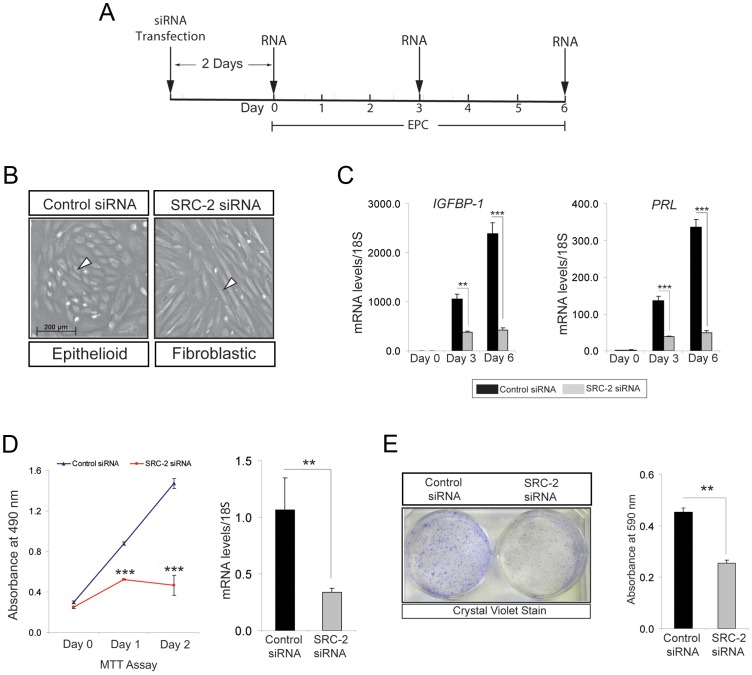
Proliferation of hESCs requires SRC-2 during the early stages of the decidual progression program. (A) Timeline for siRNA knockdown of SRC-2 in hESCs during decidualization in culture. (B) Morphology of hESCs following six days of culture in the presence of the EPC cocktail. Left panel: hESCs transfected with control (or scrambled) siRNA; right panel: hESCs transfected with siRNAs targeted to SRC-2. Note the expected polygonal (cobble stone) appearance of the epithelioid stromal cells in the presence of the control siRNA (white arrowhead). In contrast, hESCs remain fibroblastic in appearance when cultured in the presence of siRNAs to SRC-2. Scale bar in left panel applies to both panels. (C) Relative transcript levels of IGFBP-1 and PRL on day 0, 3, and 6 of EPC treated hESCs transfected with control and SRC-2 siRNA. Black and grey bars denote hESCs cultured in the presence of control and SRC-2 siRNA respectively. (D) Proliferation of control siRNA and SRC-2 siRNA transfected cells at day 1 and 2 of post EPC treatments. Error bars represent the mean standard error from triplicates. Histogram on right shows SRC-2 mRNA levels in the presence of control or SRC-2 siRNA for 48 hours in EPC media. Results are reported as mean ± SE in triplicate from one experiment. (E) Crystal violet stained hESCs transfected with control or SRC-2 siRNA after 10 days of culture in EPC cocktail (left panel). Histogram on right shows the quantitation of retained crystal violet stain from control or SRC-2 siRNA transfected hESCs. *P<0.05; **P<0.01, ***P<0.001 and ****P<0.0001.

An established *in-vitro* model for decidualization of hESCs [25], [26] was used to determine whether the early decidual defect observed in the SRC-2^d/d^ mouse translates to human (Figure 2A). While primary hESCs decidualize over a six-day culture period in response to a cocktail of E2, the progestin: MPA, and cAMP (referred hereon as EPC), small interfering (si) RNA mediated knockdown of SRC-2 levels results in an early block in this decidual response (Figure 2B, C). Unlike control cells, hESCs with reduced SRC-2 levels fail to transform from a fibroblastic to an epithelioid morphology that typifies full hESC decidualization (Figure 2B). This difference in morphological response is reflected at the molecular level by a significant attenuation in the induction of the decidual differentiation markers: prolactin (*PRL*) and insulin-like growth factor binding protein-1 (*IGFBP-1*) when SRC-2 levels are low (Figure 2C). Induction of a subset of P4 responsive decidual genes (*i.e. WNT-4* and *HAND2*) also is reduced with SRC-2 knockdown (Figure S2B). Because the hESC decidual defect occurs as early as day-3 following administration of the hormone decidual stimulus (Figure 2C), we reasoned that, like in the mouse (Figure 1), SRC-2 is required for early hESC proliferation that precedes decidualization. Indeed, MTT assays reveal that early proliferation of hESCs (as early as day 1 and 2 following exposure to the deciduogenic hormone stimulus) is markedly curtailed with SRC-2 knockdown (Figure 2D). A pro-proliferative role for SRC-2 in early decidualization is further supported by a clonogenic assay which demonstrates that reduced levels of SRC-2 limits hESC colony expansion (Figure 2E). Noteworthy, levels of SRC-1 and SRC-3 are unaffected by SRC-2 knockdown (Figure S2A) and reduction in the levels of either one of these coregulators fails to block hESC decidualization (Figure S3). Collectively, these findings highlight an evolutionary conserved and selective role for SRC-2 in early E2/P4-dependent ESC proliferation which is indispensable for the initiation of the decidual progression program.

### Endometrial stromal cell decidualization requires SRC-2 dependent glycolysis

The early requirement for SRC-2 in the decidual progression program suggests that this coregulator controls a fundamental cellular process which is critical for a quiescent ESC to rapidly proliferate in response to E2/P4. To meet the bioenergetic and biosynthetic demands of rapid cell division, the rate of gycolysis (or glycolytic flux) of a cell must be increased to generate sufficient amounts of energy (ATP) and biomass (intermediates of glucose metabolism, such as isocitrate and succinate) to enable the formation of two daughter cells [27]–[31]. Because SRC-2 belongs to a coregulator family with wide-ranging metabolic functions [19], [32], particularly in regulating the metabolic fate of glucose [18], [33], we assessed whether SRC-2 controls the glycolytic flux that underpins E2/P4-dependent ESC decidualization.

**Figure 3:**
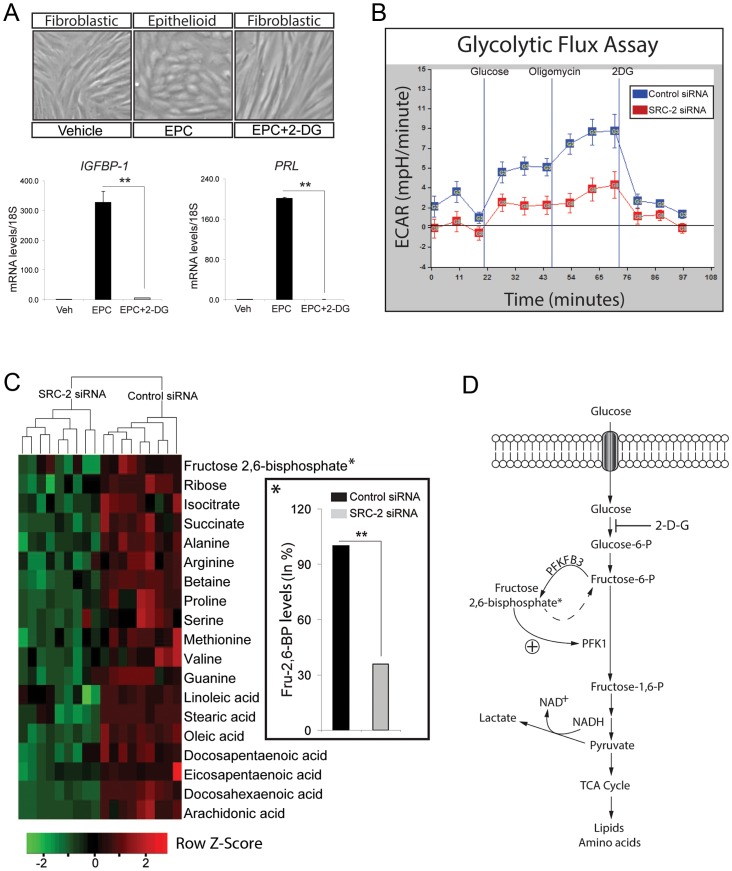
SRC-2 dependent acceleration of the glycolytic flux is required for hESC decidualization. (A) hESC decidualization following six days of culture in EPC cocktail with or without 2-DG (50 mM concentration). Top panels show the morphological changes of hESCs following the indicated treatments. Histograms at bottom display relative transcript levels of IGFBP-1 and PRL following indicated treatments. (B) Glycolytic flux assay of hESCs previously transfected with control or SRC-2 siRNA and cultured for 24 hrs in EPC (see Methods section). (C) Heat map displays metabolites that are significantly altered with SRC-2 knockdown as assessed by LC-MS analysis. Prior to LC-MS analysis, hESC lines derived from three subjects were transfected with either control or SRC-2 siRNA in triplicate and cultured in EPC media. Inset shows the significant percentage decrease in intracellular levels of fructose 2, 6-bisphosphate with SRC-2 knockdown. (D) Schematic shows the position of PFKFB3 and fructose 2, 6-bisphosphate in the glycolytic pathway.

To first confirm that glycolysis is critical for hESC decidualization, hESCs were treated with EPC in the presence or absence of 2-DG, a potent inhibitor of glucose hexokinase. As the first rate-limiting step in glycolysis, glucose hexokinase converts glucose to glucose-6-phosphate. As shown in Figure 3A, administration of 2-DG blocks hESC decidualization at the cellular and molecular level. With the inhibition of glycolysis, hESCs fail to undergo the typical fibroblastic to epithelioid cellular transformation that normally accompanies decidualization. This cellular phenotype is reflected at the molecular level by a block in the induction of the decidual biomarkers: IGFBP-1 and PRL (Figure 3A). Considering the importance of glucose uptake and utilization to decidualization along with the reported role of SRC-2 in energy homeostasis [17], [18], [34]–[37], we next asked whether SRC-2 is required for hESC glycolysis. To address this question, we employed a glycolysis stress test which measures the rate of conversion of glucose to lactate (glycolysis) non-invasively and in real time. The principle of the assay is based on the fact that cells produce and expel protons into the extracellular medium as a result of the conversion of glucose to lactate. In the first step of the assay, the rate of glycolysis (glycolytic flux) is measured by evaluating the rate of acidification of the surrounding medium (or *E*xtracellular *A*cidification *R*ate (ECAR)). In the second step of the assay, the rate of glycolysis is further increased by blocking oxidative phosphorylation using oligomycin (an ATP synthase inhibitor). With oxidative phosphorylation blocked, cells resort to “ramping up” the rate of glycolysis to maintain ATP levels and energy homeostasis (termed Glycolytic Capacity). In the assay's final step, cells are treated with 2-DG which results in a block in glycolysis as measured by a drop in ECAR; this step confirms that ECAR is solely generated by glycolysis. Applying this assay to hESCs treated with EPC in the presence or absence of siRNAs to SRC-2 (Figure 3B), we demonstrate that decreased levels of SRC-2 markedly attenuate both the glycolytic flux and glycolytic capacity of the hESC. These results strongly support an important role for SRC-2 in maintaining hESC energy homeostasis through an increase in the glycolytic flux.

Liquid chromatography-tandem mass spectrometry (LC-MS) of cell lysates from hESCs treated with EPC in the presence or absence of siRNAs to SRC-2 revealed significant differences in the output of intermediate metabolites which includes essential sugars, amino acids and fatty acids directly or indirectly derived from glycolysis (Figure 3C). Noteworthy, fructose 2, 6-bisphosphate (Fru-2, 6-BP) is significantly decreased when cellular levels of SRC-2 are reduced (Figure 3C (inset)). Generated by the bifunctional 6-phosphofructo-2-kinase/fructose-2, 6-bisphosphatase 3 (PFKFB3) [38], Fru-2, 6-BP acts as a key allosteric activator of 6-phosphofructo-1-kinase (PFK-1), a critical rate-limiting enzyme of glycolysis which converts fructose-6-phosphate to fructose-1, 6-bisphosphate [39] (Figure 3D). Based on these findings, we posit that SRC-2 may control the glycolytic flux of the hESC through regulation of intracellular levels of Fru-2, 6-BP.

### Steroid Receptor Coactivator-2 promotes hESC decidualization by sustaining induction levels of PFKFB3 expression

**Figure 4:**
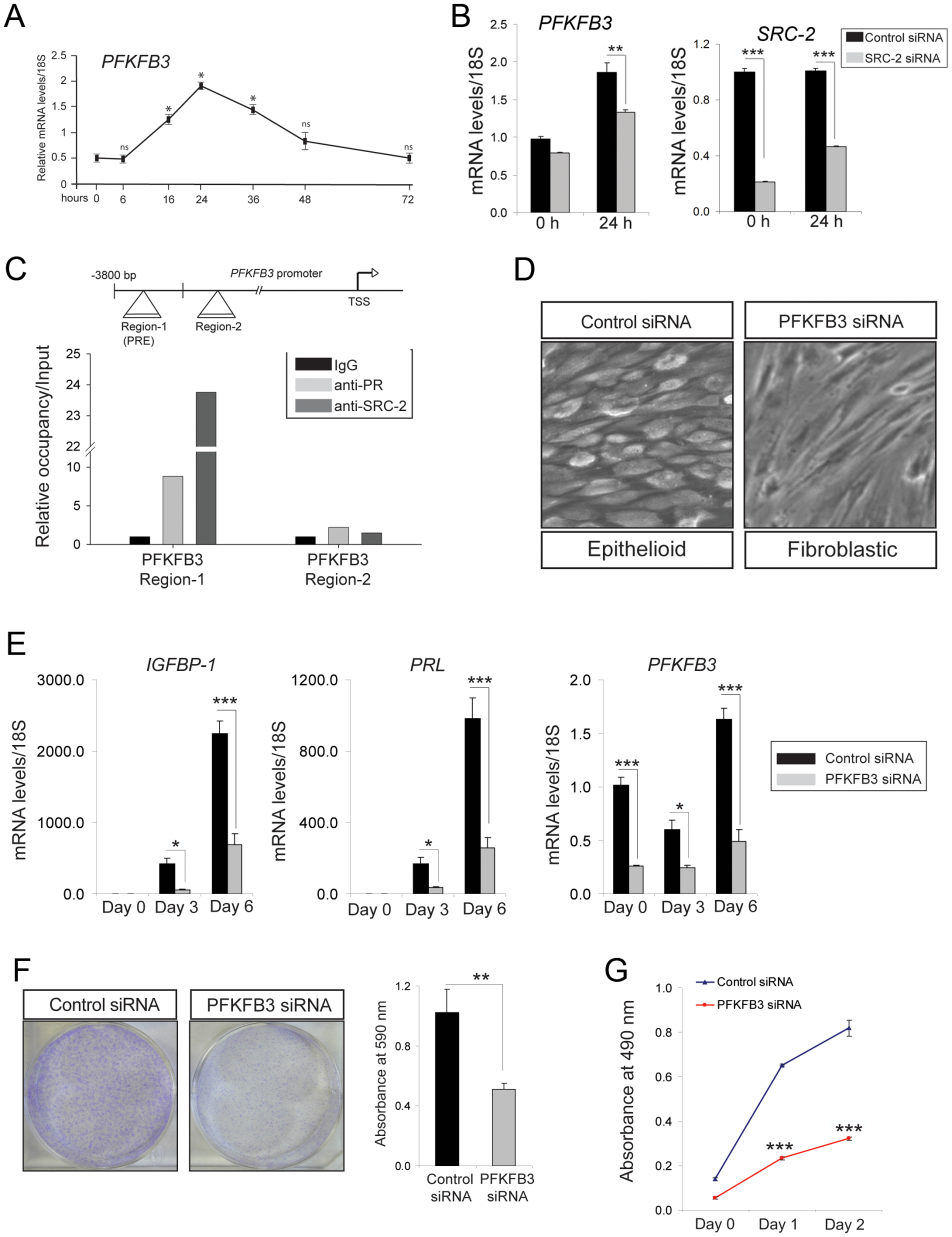
Decidualization of hESCs requires PFKFB3. (A) Real-time PCR shows the rapid induction profile of PFKFB3 in hESCs cultured in the presence of EPC cocktail for the indicated time points. (B) Relative mRNA levels of PFKFB3 and SRC-2 in hESCs transfected with control or SRC-2 siRNA and cultured in the presence of EPC cocktail for the indicated time points. (C) Schematic depicts the reported position of a PR binding site in PFKFB3 promoter (region 1 ([49])); region 2 denotes a non-binding region (negative control) for PR (TSS indicates transcriptional start site). Histogram below shows the real-time PCR result following ChIP of PR or SRC-2 on region 1 and 2. (D) Morphology of hESCs transfected with control or PFKFB3 siRNA following six days of culture in the presence of the EPC cocktail. (E) Transcript levels of IGFBP-1, PRL and PFKFB-3 in hESCs transfected with control or PFKFB3 siRNA and cultured in the presence of EPC cocktail at indicated time points. (F) Crystal violet stained control siRNA or PFKFB3 siRNA transfected hESCs after 10 days of culture in EPC cocktail (left panels). Quantitation of retained crystal violet stain from control siRNA or PFKFB3 siRNA transfected hESCs is displayed by histogram. (G) A cell viability assay (MTT) of hESCs transfected with control and PFKFB3 siRNA and cultured for the indicated time points. ****P<0.0001.

To determine whether SRC-2 controls Fru-2, 6-BP levels through regulating the expression of PFKFB3, transcript levels of PFKFB3 were measured in EPC treated hESCs in the presence or absence of siRNAs to SRC-2. Transcript levels of PFKFB3 were induced in response to EPC during the early stages of hESC decidualization (Figure 4A). However, induction of PFKFB3 during this period is reduced when SRC-2 levels are attenuated (Figure 4B). Along with the progesterone receptor, ChIP assay revealed that SRC-2 can occupy a PRE containing region within the PFKFB3 promoter (Figure 4C). Together these data suggest that SRC-2 is required for the induction of PFKFB3 by EPC through a direct transcriptional regulatory mechanism. Interestingly, however, our unpublished microarray studies on the murine uterus suggest that SRC-2 regulates not only the transcription of P4 responsive genes (such as Pfkfb3) important for stromal decidualization but also uterine genes not previously reported to be controlled by P4, indicating a broader coregulator role for SRC-2 in this tissue. To establish the predicted importance of PFKFB3 in hESC decidualization, siRNA mediated PFKFB3 knockdown was applied to EPC treated hESCs in culture for six days. At the cellular level, knockdown of PFKFB3 levels blocked the transformation of hESCs from a fibroblastic to an epithelioid phenotype, the cellular change indicative of decidualization (Figure 4D). The dependency of hESC decidualization on PFKFB3 levels was confirmed at the molecular level by a marked attenuation in the induction of the decidual markers, PRL and IGFBP-1 (Figure 4E). Importantly, clonogenic and MTT assays demonstrated that reduction in PFKFB3 levels attenuated hESC decidualization by reducing the ability of these cells to rapidly proliferate in response to EPC (Figure 4F and G).

### Pharmacological inhibition of PFKFB3 significantly attenuates ESC decidualization *in vitro* and *in vivo*

**Figure 5:**
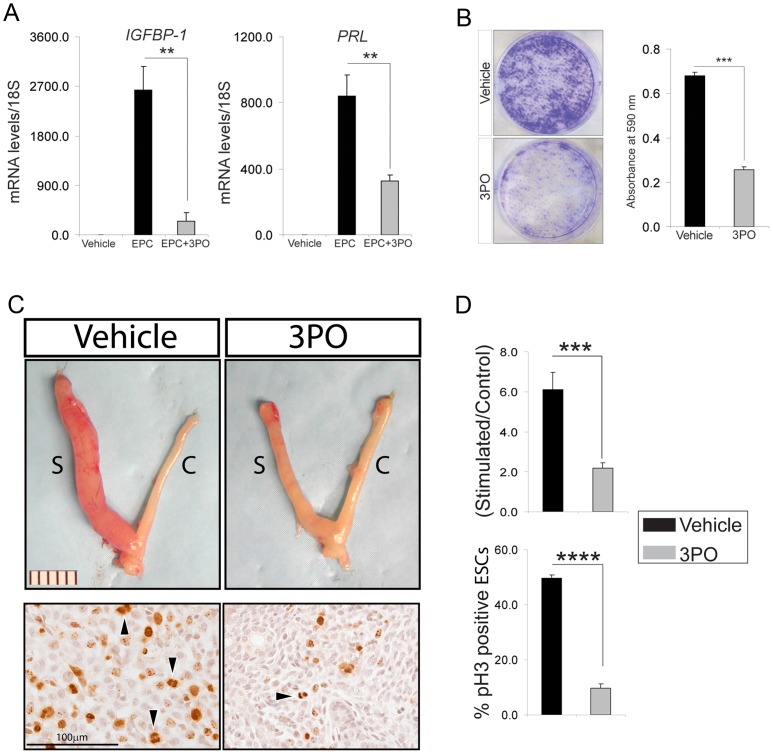
Pharmacological inhibition of PFKFB3 markedly attenuates ESC decidualization. (A) Relative transcript levels of IGFBP-1 and PRL in hESCs cultured for 6 days in the presence or absence of 3PO. (B) Crystal violet stained hESCs previously cultured in EPC in the presence of vehicle or 3PO. (C) Gross morphology of uteri exposed to vehicle or 3PO from hormone treated mice two days following receipt of the deciduogenic stimulus (See Methods and Figure S1 for further details). Scale bar applies to both panels; “S” and “C” refer to stimulated and control horn respectively. Respective panels below show representative immunohistochemical data for pH3 immunoreactivity of stimulated horns from mice treated with vehicle (left panel) or 3PO (right panel). Arrowhead denotes ESCs immunopositive pH3; scale bar applies to both panels. (D) Top histogram displays the weight-wet ratios of stimulated over control uterine horn for vehicle and 3PO treated mice which were hormone-treated and stimulated with a deciduogenic stimulus. Bottom histogram shows percentage of ESCs immunopositive for pH3 in the stimulated uterine horn of vehicle and 3PO treated mice. **P<0.01; ***P<0.001; and ****P<0.0001.

The above siRNA studies were further supported by using a small molecule inhibitor (3-(3-pyridinyl)-1-(4-pyridinyl)-2-propen-1-one (3PO) [40]) to the kinase activity of PFKFB3. In the presence of 3PO, hESCs failed to decidualize as evidenced by a marked reduction in the induction of IGFB-1 and PRL in EPC treated hESCs (Figure 5A). These results provided strong support for the proposal that the kinase activity of PFKFB3, which converts fructose 6-phosphate to fructose 2, 6-bisphosphate, is required for ESC decidualization. Because previous studies have shown that PFKFB3 is required for rapid proliferation of normal and neoplastic cells [41], [42], we reasoned that PFKFB3 may mediate SRC-2's role in early hESC proliferation. In support of this proposal, both clonogenic and MTT assays confirmed that PFKFB3 is essential for early hESC proliferation (Figure 5B and Figure S4). Together, these studies provided strong support for the proposal that PFKFB3 promotes early hESC proliferation through its kinase catalytic domain. Importantly, *Pfkfb3* also is induced in the mouse uterus by P4 which is dependent on SRC-2 and PR expression (Figure S5A). Therefore, to provide *in vivo* support for conclusions drawn from the above hESC culture studies, systemic administration of 3PO was included into the standard protocol to elicit a decidual response in ovariectomized mice (Figure S5B). Examination of uterine gross morphology two days following the administration of the deciduogenic stimulus clearly revealed that 3PO significantly attenuates the decidual response in 3PO treated mice as compared to vehicle treated controls (Figure 5C and D). Significantly, a marked decrease in ESC proliferation is observed with 3PO administration (Figure 5C and D), suggesting Pfkfb3 kinase activity also is essential for the early proliferative stages of the murine decidual progression program, which is crucial for a full decidual response (Figure S5C–F).

## Discussion

Previous pulse-labeling experiments with rodents and more recent clinical studies provide compelling support that decidual cells of the endometrium derive from highly proliferating ESCs [7], [43], [44]. From a metabolic perspective, a high rate of cellular proliferation requires a substantial increase in both glucose uptake and utilization not only to provide bioenergy but to furnish metabolic intermediates (amino acids, fatty acids, and nucleotides) to double biomass so that two daughter cells are generated following mitosis [27]–[31]. To fulfill the bioenergetic and biosynthetic demands of increased cell proliferation, the rate of glycolysis from glucose to lactate (the glycolytic flux) must be increased to rapidly furnish ATP and the necessary glycolytic intermediates to support anabolic reactions which lead to cell growth to enable mitosis. Therefore, as long as glucose is abundant, acceleration of the rate of glycolysis can provide levels of ATP and metabolic intermediates that exceed those generated by oxidative phosphorylation. Despite growing support for a critical role for enhanced glucose use in endometrial decidualization [34]–[37], [45]–[47], the key regulatory signals that direct the metabolic fate of this carbon source during the early proliferative stages of decidualization are unclear. From a clinical standpoint, identification of these regulatory signals may provide molecular insight into the etiopathogenesis of common gynecological disorders that are causally linked with impaired metabolic homeostasis as well as furnish novel targets for the clinical diagnosis and/or treatment of these co-morbidities.

We demonstrate here that SRC-2 is indispensable for the E2/P4-induced proliferation of ESCs that precedes their differentiation to decidual cells. Because early expansion of the decidual cell population is considered a key cellular event that enables deep invasion into a hypoxic environment and further development of the conceptus within the endometrium [1], our findings highlight a critical role for endometrial SRC-2 during the early steps that lead to the establishment of the maternofetal interface. For quiescent ESCs to proliferate, we reveal that SRC-2 plays a pivotal role in accelerating the glycolytic flux within predecidual cells by sustaining the induction level of PFKFB3 expression in response to E2/P4 exposure. As an inducible homodimeric enzyme, PFKFB3 converts fructose-6-phosphate to fructose-2, 6-bisphosphate [38], [39], a signaling molecule which relieves the tonic inhibitory effects of ATP on phosphofructokinase-1 (PFK-1), a major rate-limiting glycolytic enzyme. With unrestricted acceleration of the glycolytic flux through the PFK-1 checkpoint, anabolic pathways such as the pentose phosphate pathway can be utilized to support rapid ESC proliferation and decidualization [35]. Interestingly, P4 has been reported to induce PFKFB3 in human breast cancer cells [48], [49], suggesting that PFKFB3 may mediate P4 mitogenic effects both in normal and abnormal physiologic contexts. Indeed, PFKFB3 is induced by a myriad of mitogenic, inflammatory, and hypoxic stimuli and is constitutively expressed in a number of leukemias and solid tumors [49]–[55]. Although PFKFB3 has been shown to be expressed in human placenta [56], whether deregulation of this regulatory kinase can lead to proliferative disorders of the endometrium such as endometriosis, hyperplasia, or cancer constitutes an important question for future investigation.

Modulation of the ESC glycolytic flux by SRC-2 is in keeping with an expanding role for this coregulator in glucose metabolism [17]–[19]. A member of the p160/SRC family of pleiotropic coregulators of glucose, fatty acid, and protein metabolism [19], SRC-2 has been shown to be critical for hepatic glucose release during periods of caloric restriction [18]. During persistent periods of energy insufficiency, a critical role for SRC-2 is to release glucose from residual hepatic glycogen stores to maintain survival of the individual. During periods of glucose abundance, however, we show here that SRC-2 is crucial for rapid utilization of this energy source for endometrial decidualization, an essential reproductive process for the perpetuation of the species.

In conclusion, we demonstrate that SRC-2 is essential for the metabolic reprogramming of the predecidual ESC into a proliferative phenotype, an essential early step toward endometrial decidualization. Our findings not only offer an important conceptual advance in our understanding of endometrial SRC-2 in peri-implantation biology but may well provide mechanistic underpinnings to explain the role of this coregulator in other areas of endometrial physiology (*i.e.* preterm labor [14]) as well as in endometrial pathophysiologies in women diagnosed with leiomyoma or polycystic ovary syndrome [13], [16]. Finally, our new findings furnish the pretext for considering SRC-2 and its metabolic targets in the future design of new clinical approaches to more effectively diagnose and/or treat a non-receptive uterus in women with recurrent implantation failure or early pregnancy loss.

## Methods

### Mice and hormone treatments

The *PR* ^Cre/+^ *SRC*-2^f/f^ (*SRC*-2^d/d^) bigenic mouse was generated by crossing our *PR* ^Cre/+^ knock-in [57] with the *SRC*-2^f/f^ mouse (TIF2 floxed [L2 version]) [58]. In the SRC-2^d/d^ bigenic, SRC-2 is abrogated specifically in progesterone receptor (PR) positive cells [12]. In a 12-hour light: 12-hour dark recurrent cycle, mice were housed within a *vivarium* maintained at Baylor College of Medicine. Mice received standard rodent chow and water *ad libitum* and were humanely treated in accordance with institutional guidelines for animal care and use (IACUC). For the hormone-induced uterine receptivity model (Figure 1A and [20]), eight week-old mice were ovariectomized before priming two weeks later with two daily injections of E2 (100 ng/day/mouse; Sigma-Aldrich, St. Louis, MO). Following two days of rest, mice were administered one of the following hormone treatments: 1) four daily injections of sesame oil (E2 primed); 2) three daily injections of sesame oil followed by an injection of E2 (50 ng) on day 4 (nidatory E2); or 3) three daily injections of 1 mg of P4 (Sigma-Aldrich) followed on day 4 by an injection of 1 mg P4 plus 50 ng E2 (E2P4). Fifteen hours following the final hormone injection, mice were intraperitoneally (I.P.) injected with 5′-bromo-2′-deoxyuridine (BrdU) (Amersham Biosciences Corporation, Piscataway, NJ (0.1 ml/10 g of body weight)) two hours before sacrifice.

To elicit an artificial decidual response [9], mice were ovariectomized at six weeks-of-age and received three daily injections of E2 (100 ng) two weeks following ovariectomy (see: Figure S1A). After 2 days of rest, mice received three daily injections of E2P4 (E2 (6.7 ng) plus P4 (1 mg)). Six hours after the third E2P4 injection, intraluminal instillation of 50 µl of sesame oil was performed on the left uterine horn (stimulated); the right horn was not stimulated (control). After oil instillation, mice received daily injections of E2 (6.7 ng) plus P4 (1 mg) for either 2 days or 5 days before sacrifice (Figure 1E and Figure S1 respectively). Uterine tissue was collected for wet-weight measurement as well as for histological and molecular analysis.

### Histological analysis

Uterine tissue was fixed in 4% paraformaldehyde (PFA) overnight at 4°C, dehydrated through graded ethanol washes, embedded in paraffin wax before being sectioned into 5 µm thick tissue sections onto Superfrost Plus glass slides (Fisher Scientific, Pittsburgh, PA). For immunohistochemical analysis, tissue sections were deparaffinized, rehydrated and boiled in a citric acid based antigen unmasking solution (Vector laboratories Inc., Burlingame, CA). After blocking, sections were incubated with a rabbit polyclonal anti-phospho-histone H3 antibody (EMD Millipore, Billerica, MA) overnight at 4°C. After the primary antibody incubation, sections were incubated with goat anti-rabbit IgG secondary antibody (Vector laboratories Inc.) for 1 hour at room temperature followed by an incubation with ZyMax streptavidin-horse radish peroxidase conjugate (Invitrogen Corporation, Carlsbad, CA) for 30 minutes at room temperature. Immunoreactivity was visualized with the 3, 3′-diaminobenzidine (DAB) peroxidase substrate kit (Vector laboratories Inc.) and counterstained with hematoxylin. Finally, sections were dehydrated and mounted using permount histological mounting medium (Fisher Scientific Inc.).

For BrdU immunohistochemical analysis, uterine sections were fixed, processed, embedded, and sectioned as above. Following blocking, sections were incubated with a biotinylated anti-BrdU antibody (BrdU In-Situ Detection Kit (BD Pharmingen Inc., San Jose, CA)) overnight at room temperature. Tissue sections were then incubated with vectastain ABC reagent (Vector laboratories Inc.) at room temperature for 1 hour and developed with the DAB peroxidase substrate kit. Finally, sections were counterstained with hematoxylin and cover slipped.

### Quantitative real-time PCR

For quantitative real-time PCR, total RNA from mouse uteri or hESCs (see below) was isolated using the RNeasy total RNA isolation kit (Qiagen Inc., Valencia, CA). Total RNA was reverse transcribed to cDNA using the TaqMan reverse transcription kit (Applied Biosystems, Foster City, CA). The PCR conditions used for cDNA template synthesis were 10 minutes at 25°C, 30 minutes at 48°C, and 5 minutes at 95°C. Quantitative real-time PCR analysis was performed using TaqMan 2× master mix along with validated primers (Applied Biosystems). Resultant amplicons were detected and quantitated using an ABI Prism 7700 sequence detection system; 18S ribosomal RNA was used as an internal control; gene specific primers used in these studies are listed in Table S1.

### Human endometrial stromal cell isolation and culture

Prior to endometrial tissue biopsy, written-informed consent was obtained from all participating subjects. Clinical procedures undertaken to biopsy endometrial tissue followed Institutional Review Board (IRB) protocols from Baylor College of Medicine and in accordance with the guidelines of the Declaration of Helsinki [59]. Endometrial biopsy samples were obtained during the proliferative phase of the menstrual cycle (day 7–10) of healthy women with regular menstrual cycles. Endometrial tissue was biopsied from the uterine fundus using a Pipelle catheter (Unimar, Bridgeport, CT) before being cut into small pieces using sterile scissors and subsequently digested in DMEM/F12 medium containing collagenase (2.5 mg/ml (Sigma-Aldrich)) and DNase I (0.5 mg/ml (Sigma-Aldrich)) for 1.5 hours at 37°C [26]. After digestion, dispersed cells were collected by centrifugation and layered over a Ficoll-Paque reagent layer (GE Healthcare Biosciences, Pittsburgh, PA) to remove lymphocytes. The top layer containing the hESC fraction was collected and filtered through a 40 µm nylon cell strainer (BD Biosciences, Franklin Lakes, NJ). Fractionated hESCs were then resuspended in DMEM/F-12 media containing 10% FBS, 100 units/ml penicillin and 0.1 mg/ml streptomycin (hESC media) and cultured in tissue culture flasks (75 cm^2^). Experiments were carried out using primary hESCs derived from at least three individual subjects; only early passage hESCs were used in these studies (≤ passage 4).

### Transfection and hESC decidualization

Six well culture plates were used to culture hESCs (1×10^5^ cells per well (9.5 cm^2^) in triplicate). Smart pools of siRNAs targeting PFKFB-3 (1027416 (Qiagen Inc.)), SRC-1 (NCOA-1 (L-005196-00-0005)), SRC-2 (NCOA-2 (L-020159-00-0005)), SRC-3 (NCOA-3 (L-003759-00-0005)), and or non-targeting siRNA (D-001810-10-05) (Thermo Scientific, Dharmacon RNAi Technologies, Chicago, IL) were used to transfect hESCs at 60–70% confluency. Per well, siRNAs (60 pmoles) were transfected into hESCs using Lipofectamine 2000 reagent in 1× Opti-MEM I reduced-serum media (Invitrogen Corporation). Five hours post-transfection, media was replaced with hESC media. Forty-eight hours later, hESCs were treated with 1× Opti-MEM I reduced-serum media containing 2% fetal bovine serum (FBS), E2 (100 nM), medroxyprogesterone acetate (MPA: 10 µM (Sigma-Aldrich)) and cAMP (50 µM (Sigma-Aldrich)) which constitutes the decidualization media. The first day that hESCs were cultured in decidualization media was assigned day 0. For these studies, decidualization media was renewed every two days. Cells were harvested at appropriate time points as per experimental conditions. Total RNA was isolated to assess transcript levels of the decidualization markers: prolactin (PRL) and insulin-like growth factor binding protein-1 (IGFBP-1) [25].

### Colony formation, cell viability and cell death assays

Forty-eight hours following siRNA transfection, hESCs were trypsinized and counted. For colony formation assays, 5×10^3^ cells were plated per well of six-well plates in triplicate. Cells were grown in either decidualization or hESC medium; culture media was changed every two days for 10 days [60]. After 10 days, hESCs were washed with 1XPBS and stained with Accustain crystal violet solution (Sigma-Aldrich). After staining for 15 minutes, cells were washed three times with water to remove unbound stain before plates were air-dried overnight. The following day, stained colonies were photographed and counted before the retained crystal violet stain was removed with 10% acetic acid; the stain eluate was quantitated by spectrophotometry at 490 nm. For cell viability assays, 10^3^ hESCs were plated per well of a 96-well plate (in triplicate) 48 hours following transfection with siRNA. Following adherence to the well floor, cells were treated with decidualization or hESC complete media. Cell viability was determined using the CellTiter 96 Non-Radioactive Cell Proliferation Assay (Promega Corporation, Madison, WI) per the manufacturer's instructions. For cell death assays, forty-eight hours following siRNA transfections, hESCs were trypsinized and 10^3^ hESCs were plated per well of a 96-well plate (in triplicate). Following adherence, cells were treated with decidualization or hESC complete media for seventy two hours. Cell death assay was determined using the Apo-ONE Homogeneous Caspase-3/7 Assay (Promega Corporation, Madison, WI) as per the manufacturer's instructions.

### Western blot analysis

Forty-eight hours following siRNA transfections, protein extracts were prepared from hESCs as explained previously [61]. Protein extracts were resolved on sodium dodecyl sulfate-polyacrylamide gel electrophoresis and transferred on to polyvinylidene difluoride membranes (Bio-Rad Laboratories, Hercules, CA). Polyclonal anti-rabbit SRC-2 (Bethyl laboratories, Montgomery, TX), Polyclonal anti-rabbit PFKFB3 (Cell Signaling, Danvers, MA) and a monoclonal anti-mouse β-actin (Sigma-Aldrich) antibodies were used for detecting SRC-2, PFKFB3 and β-actin proteins respectively. Appropriate horseradish peroxidase-conjugated immunoglobulin G secondary antibodies (Santa Cruz Biotechnology, Santa Cruz, CA) were used to amplify the primary antibodies signal and immunoreactive bands were visualized by using enhanced chemiluminescence substrate detection kit (Pierce Biotechnology, Rockford, IL).

### Chromatin immunoprecipitation assays

Chromatin immunoprecipitation (ChIP) assays were performed using the ChIP-IT Express kit (ActiveMotif Inc., Carlsbad, CA). Briefly, hESCs were cultured to 70–80% confluency before fixation with 10% formaldehyde for 10 minutes. After fixation, cells were lysed before being homogenized using a dounce homogenizer to obtain the nuclear fraction. Nuclei were resuspended in chromatin shearing buffer and subjected to sonication to fragment the chromatin. Fragmented chromatin was immunoprecipitated overnight either with a rabbit polyclonal antibody specific to the human progesterone receptor ((H-190) Santa Cruz Biotechnology Inc., Santa Cruz, CA) or human SRC-2 (Bethyl Laboratories, Montgomery, TX). Incubation with a rabbit polyclonal IgG antibody (Santa Cruz Biotechnology Inc.) served as a control for non-specific immunoprecipitation. After immunoprecipitation, fragmented chromatin was reverse cross-linked before elution and purification of immunoprecipitated DNA fragments. Quantitative real-time PCR was performed using these DNA fragments as templates to determine the occupancy of SRC-2 and PR at two distinct regions (termed region 1 and 2) within the human PFKFB-3 promoter [49].

### Glycolytic flux analysis

Glycolytic flux analysis was performed using the XF Glycolysis Stress Kit with the Seahorse Biosciences XF analyzer as per the manufacturer's protocol (Seahorse Bioscience, North Billerica, Massachusetts [39]). Forty-eight hours following siRNA transfection, hESCs were trypsinized and 1×10^4^ cells were plated per well of 24-well plates as five replicates. After 24 hours of EPC treatment, media was replaced with assay media and incubated at 37°C for 1 hr to equilibrate the media pH prior to assay. After equilibration, three well-defined small molecule modulators of glycolysis: glucose and oligomycin (both promoters of glycolysis) and 2-Deoxy-D-glucose (2DG) (a glycolysis inhibitor) were sequentially administered. Glycolysis leads to proton extrusion from the cell which results in the acidification of surrounding media. This *E*xtra *C*ellular *A*cidification *R*ate (ECAR) was measured at specific time points to assay and profile the glycolytic flux in real time. Determined ECAR rates were plotted versus time after the administration of glucose, oligomycin, and 2DG into the assay media (error bars represent the standard mean error of biological replicates).

### Metabolic profiling

Control or SRC-2 siRNA were transfected into hESCs derived from three different subjects in triplicate as explained above. Forty-eight hours following siRNA transfection, hESCs were treated with EPC cocktail. Cell pellets were prepared from hESCs following twenty-four hours of culture and subjected to liquid chromatography tandem mass spectrometry (LC-MS) analysis to determine the levels of intracellular metabolites [62]. As a positive control, a pool of mouse liver tissue was included in the LC-MS analysis. For each subject, the data from each group of metabolites (amino acids, sugars, and fatty acids) were normalized separately using the internal standard. Each data-set was log_2_ transformed and used to calculate the median based fold change relative to control samples. Heat maps were created using log transformed data to highlight the differences between the control siRNA and SRC-2 siRNA transfected cells. For the hierarchical classification, the average linkage based hierarchical classification was performed using the scaled log transformed data.

### Statistical analysis

Statistical analyses of data were performed using one-way ANOVA followed by Tukey's post hoc multiple range test and two-tailed Student's *t* test with the Instat Tool package version 3.0 (GraphPad software Inc., La Jolla, CA); results were considered statistically significant with a *P* value<0.05.

## Supporting Information

Figure S1 Murine SRC-2 is indispensable for steroid hormone-induced ESC decidualization. (A) Time-line for induction of the artificial deciduogenic response (Methods and [9]). Note: data shown in (B) and (C–F) corresponds to tissue analyzed two and five days following the deciduogenic stimulus (oil instillation) respectively. These time points are indicated by (2) and (5) in time-line schematic. (B) Quantitative real-time PCR analysis of transcript levels for Wnt4, Hand2, and Bmp2 in the control and stimulated horn from SRC-2^f/f^ and SRC-2^d/d^ mice. (C) Gross morphological response of uteri from SRC-2^f/f^ and SRC-2^d/d^ mice five days following the deciduogenic stimulus. Scale bar applies to both panels. (D) Weight-wet ratios of stimulated (S) over control (C) uterine horn from SRC-2^f/f^ and SRC-2^d/d^. (E) Hematoxylin and eosin stained sections of stimulated horns from SRC-2^f/f^ (top panel) and SRC-2^d/d^ (bottom panel). Arrowhead indicates a large polygonal epithelioid cell and a small undifferentiated ESC in the top and bottom panels respectively (scale bar applies to both panels). (F) Quantitative real-time PCR analysis of Bmp2 and Wnt4 transcript levels in stimulated and control uterine horns from SRC-2^f/f^ and SRC-2^d/d^ mice. Relative transcript levels were quantitated by comparing with the levels of SRC-2 f/f mice control horn. Results represent means ±SE; n = 5 mice/group. *P<0.05; **P<0.01.
(TIF)

Figure S2 Expression levels of SRC-1 and SRC-3 are not altered with siRNA knockdown of SRC-2 in hESCs. (A) Transcript levels for SRC-1, SRC-2 and SRC-3 in control siRNA or SRC-2 siRNA transfected hESCs at day 0, 3 and 6 of post EPC treatments. (B) Transcript levels for progestin responsive genes, WNT4 and HAND2 in control or SRC-2 siRNA transfected hESCs at day 0, 3 and 6 of post EPC treatments. (C) Western blot analysis of SRC-2 protein levels to determine the effectiveness of SRC-2 siRNA from hESCs transfected with control siRNA or SRC-2 siRNA at 48 hours post transfections. HepG2 cell lysate was used as positive control and β-actin was used as loading control.
(TIF)

Figure S3 Decidualization of hESCs does not require SRC-1 or SRC-3. (A) Transcript levels of IGFBP-1 and PRL in hESCs transfected with control siRNA or SRC-1 siRNA or SRC-3 siRNA at day 0, 3 and 6 of post EPC treatments. (B) Transcript levels of SRC-1, SRC-2 and SRC-3 in hESCs transfected with control siRNA or SRC-1 siRNA or SRC-3 siRNA during indicated time points of hESCs decidualization.
(TIF)

Figure S4 (A) Western blot analysis of PFKFB3 protein levels from hESCs transfected with control siRNA or PFKFB3 siRNA at 48 hours post transfections. HepG2 cell lysate was used as positive control and β-actin was used as loading control. (B) A cell viability assay (MTT) of hESCs cultured in the presence of EPC in the presence of vehicle or 3PO at the indicated time points. ***P<0.001. (C) Transcript levels of IGFBP-1, PRL and PFKFB-3 in hESCs transfected with control or PFKFB3 siRNA and cultured in the presence of EPC cocktail at day 9 of post EPC treatments. (D) Measurement of active caspase-3/7 in hESCs transfected with control or PFKFB3 siRNA and cultured in the presence of vehicle or EPC cocktail at 72 hours post EPC treatments. Active caspase-3/7 enzymatic activity is represented as relative fluorescent units in percent relative to vehicle treated control siRNA transfected cells. (E) Active caspase-3/7 enzymatic activity in hESCs following three days of culture in EPC cocktail with or without 2-DG (50 mM concentration). Active caspase-3/7 enzymatic activity is represented as relative fluorescent units in percent relative to vehicle treated cells.
(TIF)

Figure S5 Murine endometrial decidualization requires Pfkfb3. (A) Comparative transcript levels of Pfkfb3 in uteris derived from SRC-2^f/f^; SRC-2^d/d^; and progesterone receptor knockout (PRKO) mice treated for 6-hours with P4 (1 mg). Results represent means ± SE; n = 4 mice/group with ***P<0.001. (B) Time-line for induction of the artificial deciduogenic response in the presence or absence of 3PO. (C) Low power magnification of a transverse section of the stimulated uterine horn stained for pH3 immunoreactivity from wild type mice two days following the deciduogenic stimulus and previously treated with DMSO (vehicle) as indicated in the above time-line. (D) Stimulated uterine horn stained for pH3 immunoreactivity from wild type mice similarly treated with the deciduogenic stimulus but treated with 3PO as indicated in the above time-line; scale bar in (C) applies to (D). Panels (E) and (F) represent pH3 stained sections of stimulated horns shown in (C) and (D) respectively. Arrowhead indicates a stromal cell positive for pH3 immunoreactivity. Scale bar in (E) applies to (F).
(TIF)

Figure S6 Quantitative real-time PCR analysis of transcript levels for Ccnd3, Cdk6, Cdc25c and Cdkn1a in the control and stimulated horns following two days of deciduogenic stimulus from SRC-2^f/f^ and SRC-2^d/d^ mice. Results represent means ±SE; n = 5 mice/group. *P<0.05; ***P<0.001.
(TIF)

Table S1 List of gene specific primers used in Quantitative Real-Time PCR analysis.
(TIF)
