# Correction: Acceleration of the Glycolytic Flux by Steroid Receptor Coactivator-2 Is Essential for Endometrial Decidualization

**DOI:** 10.1371/journal.pgen.1005515

**Published:** 2015-09-29

**Authors:** Ramakrishna Kommagani, Maria M. Szwarc, Ertug Kovanci, William E. Gibbons, Nagireddy Putluri, Suman Maity, Chad J. Creighton, Arun Sreekumar, Francesco J. DeMayo, John P. Lydon, Bert W. O'Malley

The following information is missing from the Funding section: CPRIT Core Facility Support Award RP120092 "Proteomic and Metabolomics Core Facility”.

The correct funding statement is: This research was supported by grants from the National Institutes of Health and Alkek Center for Molecular Discovery (RO1 HD-07857, DK59820, HD8818, HD-042311, RO1 CA-77530, and U54 HD-0077495 to FJD, AS, JPL, and BWO), and CPRIT Core Facility Support Award RP120092 "Proteomic and Metabolomics Core Facility”. The funders had no role in study design, data collection and analysis, decision to publish, or preparation of the manuscript.
