# Supplementary figures and images for: Acceleration of the Glycolytic Flux by Steroid Receptor Coactivator-2 Is Essential for Endometrial Decidualization
Source: PLoS Genet. 2013 Oct 24;9(10):e1003900. doi: 10.1371/journal.pgen.1003900 (PMC3812085; doi:10.1371/journal.pgen.1003900)

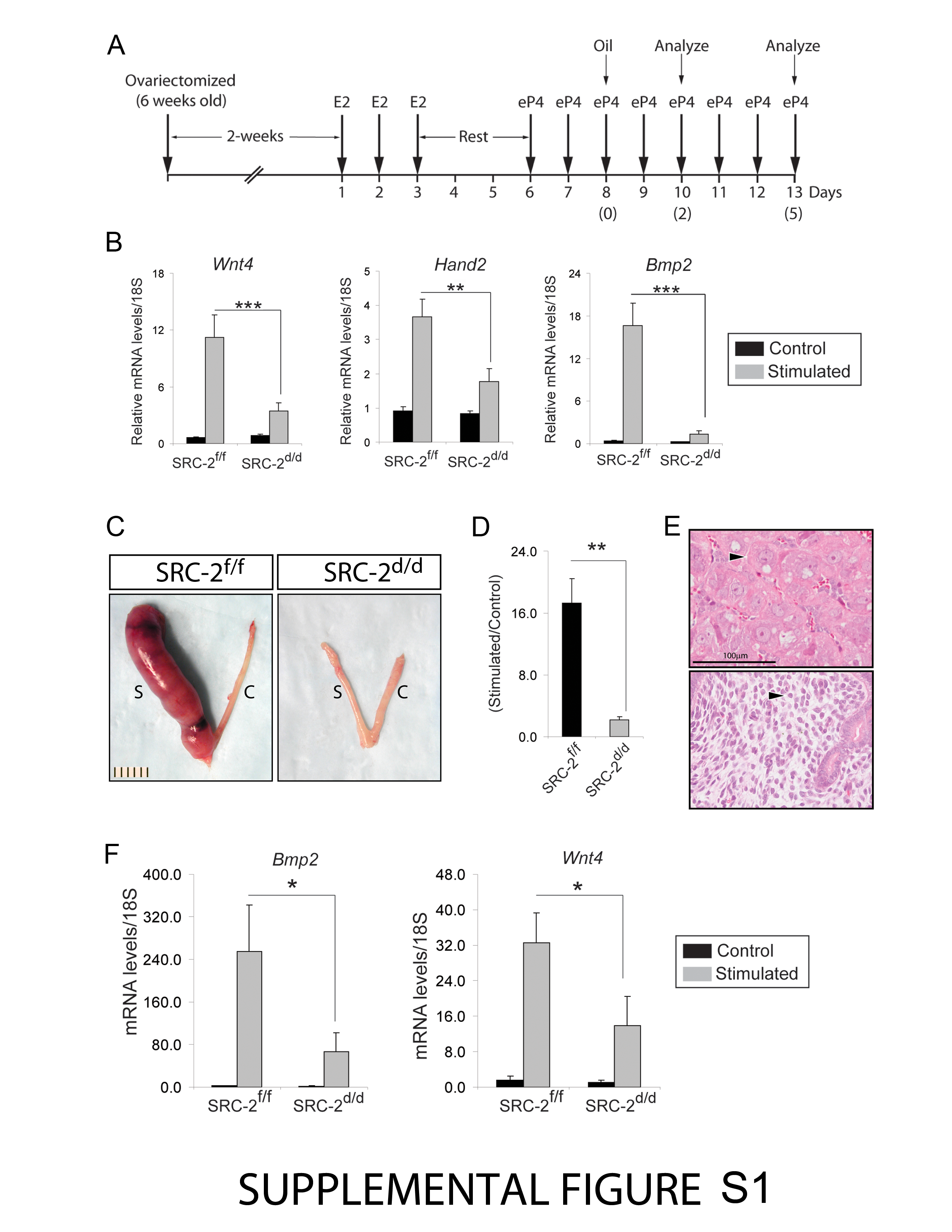

Supplement: Figure S1 — Murine SRC-2 is indispensable for steroid hormone-induced ESC decidualization. (A) Time-line for induction of the artificial deciduogenic response (Methods and [9]). Note: data shown in (B) and (C–F) corresponds to tissue analyzed two and five days following the deciduogenic stimulus (oil instillation) respectively. These time points are indicated by (2) and (5) in time-line schematic. (B) Quantitative real-time PCR analysis of transcript levels for Wnt4, Hand2, and Bmp2 in the control and stimulated horn from SRC-2f/f and SRC-2d/d mice. (C) Gross morphological response of uteri from SRC-2f/f and SRC-2d/d mice five days following the deciduogenic stimulus. Scale bar applies to both panels. (D) Weight-wet ratios of stimulated (S) over control (C) uterine horn from SRC-2f/f and SRC-2d/d. (E) Hematoxylin and eosin stained sections of stimulated horns from SRC-2f/f (top panel) and SRC-2d/d (bottom panel). Arrowhead indicates a large polygonal epithelioid cell and a small undifferentiated ESC in the top and bottom panels respectively (scale bar applies to both panels). (F) Quantitative real-time PCR analysis of Bmp2 and Wnt4 transcript levels in stimulated and control uterine horns from SRC-2f/f and SRC-2d/d mice. Relative transcript levels were quantitated by comparing with the levels of SRC-2 f/f mice control horn. Results represent means ±SE; n = 5 mice/group. *P<0.05; **P<0.01. (TIF) [file pgen.1003900.s001.tif]

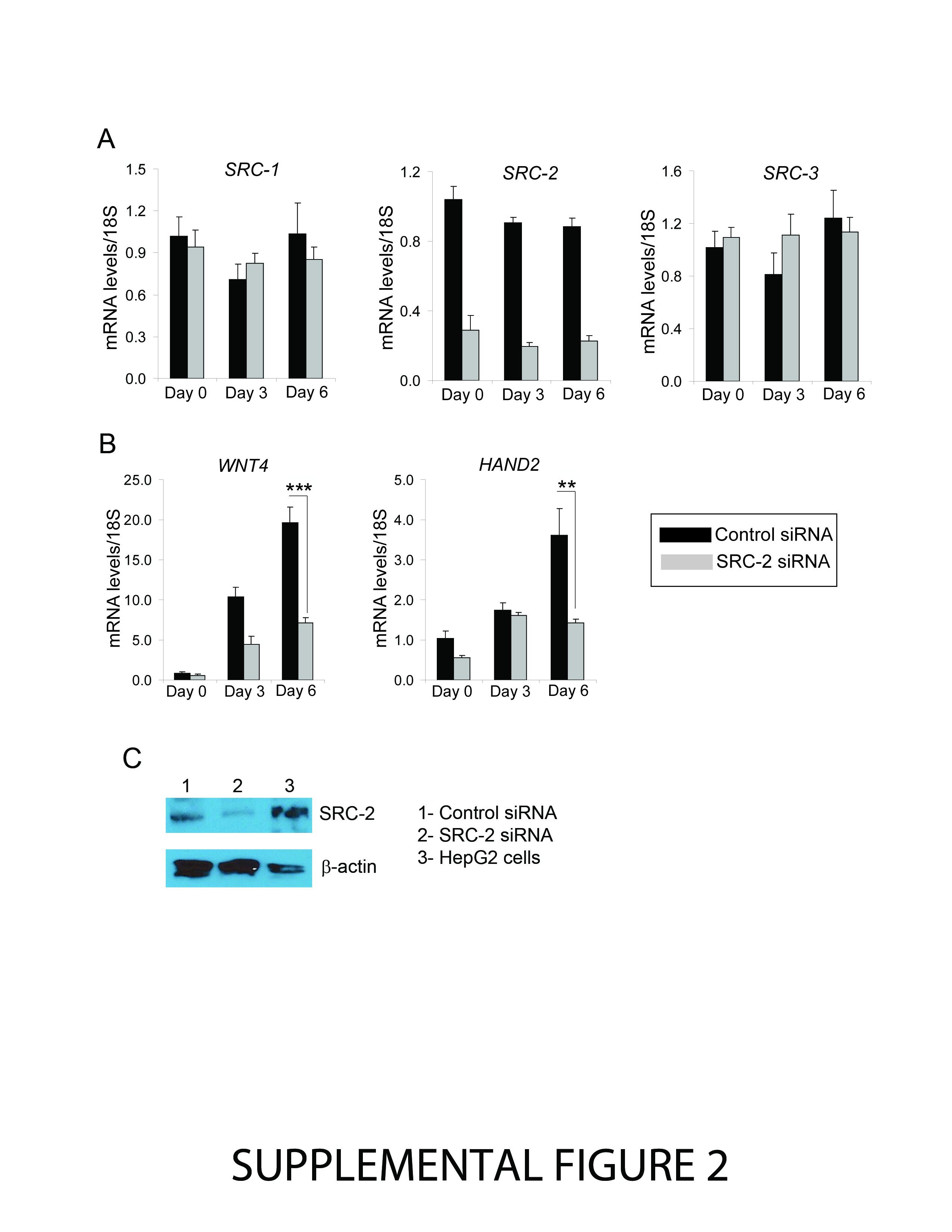

Supplement: Figure S2 — Expression levels of SRC-1 and SRC-3 are not altered with siRNA knockdown of SRC-2 in hESCs. (A) Transcript levels for SRC-1, SRC-2 and SRC-3 in control siRNA or SRC-2 siRNA transfected hESCs at day 0, 3 and 6 of post EPC treatments. (B) Transcript levels for progestin responsive genes, WNT4 and HAND2 in control or SRC-2 siRNA transfected hESCs at day 0, 3 and 6 of post EPC treatments. (C) Western blot analysis of SRC-2 protein levels to determine the effectiveness of SRC-2 siRNA from hESCs transfected with control siRNA or SRC-2 siRNA at 48 hours post transfections. HepG2 cell lysate was used as positive control and β-actin was used as loading control. (TIF) [file pgen.1003900.s002.tif]

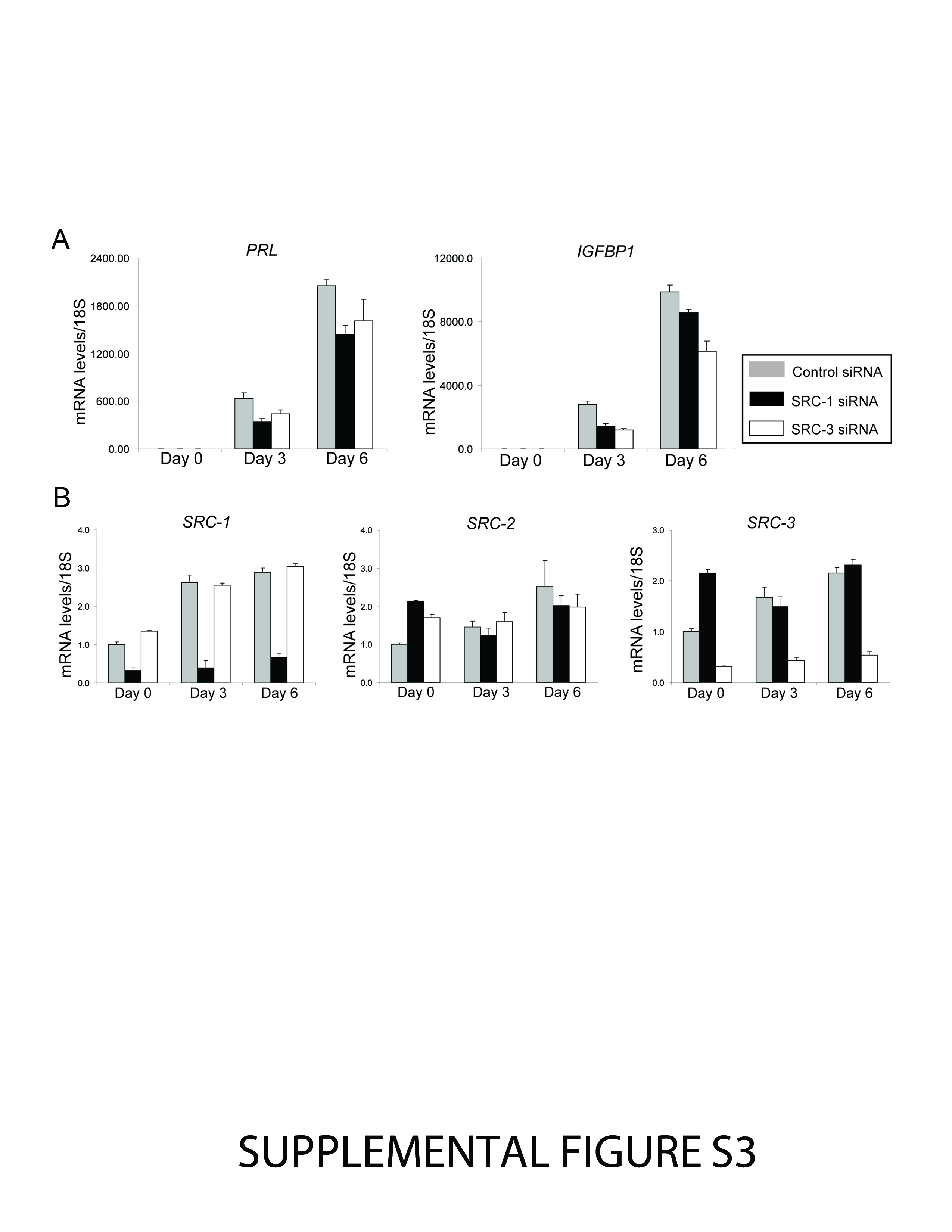

Supplement: Figure S3 — Decidualization of hESCs does not require SRC-1 or SRC-3. (A) Transcript levels of IGFBP-1 and PRL in hESCs transfected with control siRNA or SRC-1 siRNA or SRC-3 siRNA at day 0, 3 and 6 of post EPC treatments. (B) Transcript levels of SRC-1, SRC-2 and SRC-3 in hESCs transfected with control siRNA or SRC-1 siRNA or SRC-3 siRNA during indicated time points of hESCs decidualization. (TIF) [file pgen.1003900.s003.tif]

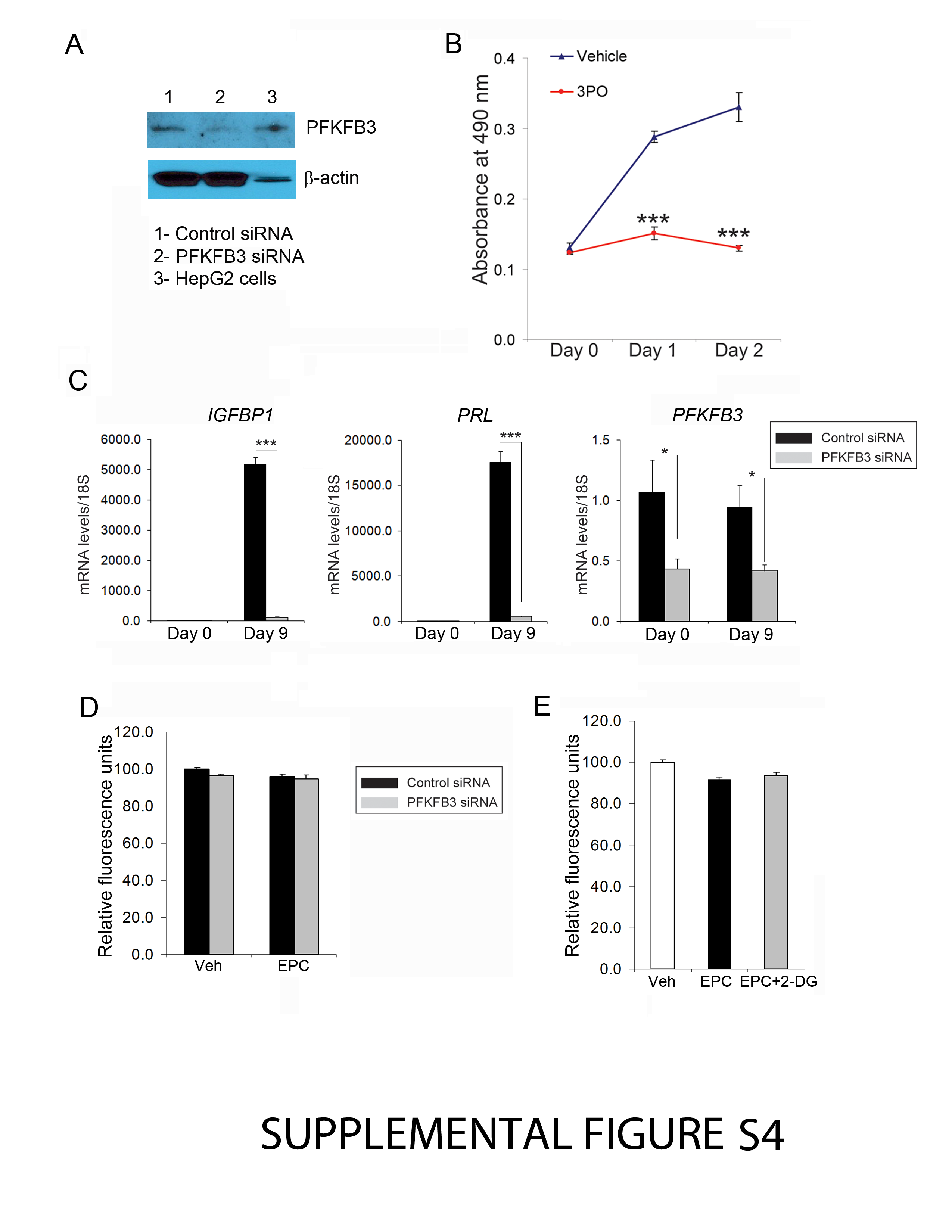

Supplement: Figure S4 — (A) Western blot analysis of PFKFB3 protein levels from hESCs transfected with control siRNA or PFKFB3 siRNA at 48 hours post transfections. HepG2 cell lysate was used as positive control and β-actin was used as loading control. (B) A cell viability assay (MTT) of hESCs cultured in the presence of EPC in the presence of vehicle or 3PO at the indicated time points. ***P<0.001. (C) Transcript levels of IGFBP-1, PRL and PFKFB-3 in hESCs transfected with control or PFKFB3 siRNA and cultured in the presence of EPC cocktail at day 9 of post EPC treatments. (D) Measurement of active caspase-3/7 in hESCs transfected with control or PFKFB3 siRNA and cultured in the presence of vehicle or EPC cocktail at 72 hours post EPC treatments. Active caspase-3/7 enzymatic activity is represented as relative fluorescent units in percent relative to vehicle treated control siRNA transfected cells. (E) Active caspase-3/7 enzymatic activity in hESCs following three days of culture in EPC cocktail with or without 2-DG (50 mM concentration). Active caspase-3/7 enzymatic activity is represented as relative fluorescent units in percent relative to vehicle treated cells. (TIF) [file pgen.1003900.s004.tif]

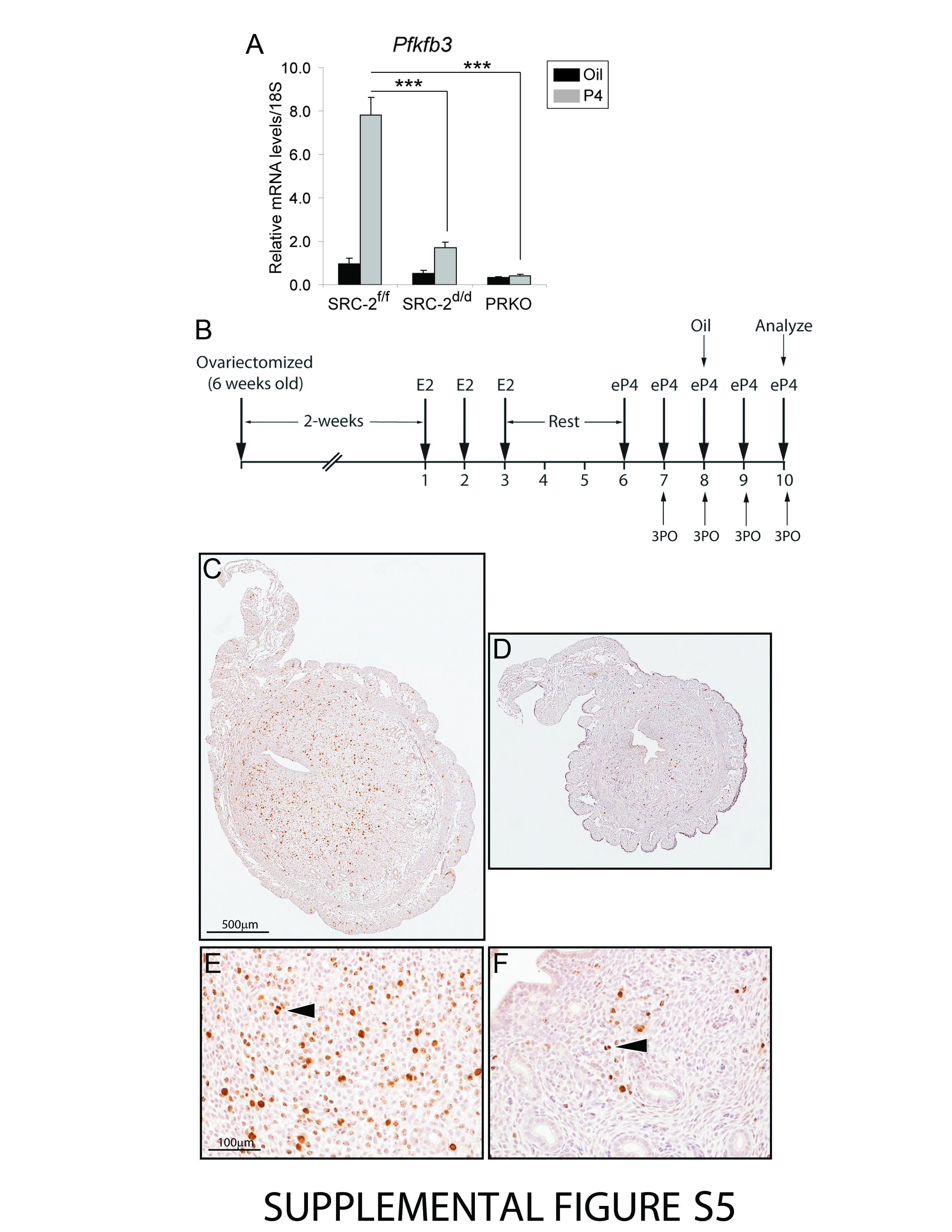

Supplement: Figure S5 — Murine endometrial decidualization requires Pfkfb3. (A) Comparative transcript levels of Pfkfb3 in uteris derived from SRC-2f/f; SRC-2d/d; and progesterone receptor knockout (PRKO) mice treated for 6-hours with P4 (1 mg). Results represent means ± SE; n = 4 mice/group with ***P<0.001. (B) Time-line for induction of the artificial deciduogenic response in the presence or absence of 3PO. (C) Low power magnification of a transverse section of the stimulated uterine horn stained for pH3 immunoreactivity from wild type mice two days following the deciduogenic stimulus and previously treated with DMSO (vehicle) as indicated in the above time-line. (D) Stimulated uterine horn stained for pH3 immunoreactivity from wild type mice similarly treated with the deciduogenic stimulus but treated with 3PO as indicated in the above time-line; scale bar in (C) applies to (D). Panels (E) and (F) represent pH3 stained sections of stimulated horns shown in (C) and (D) respectively. Arrowhead indicates a stromal cell positive for pH3 immunoreactivity. Scale bar in (E) applies to (F). (TIF) [file pgen.1003900.s005.tif]

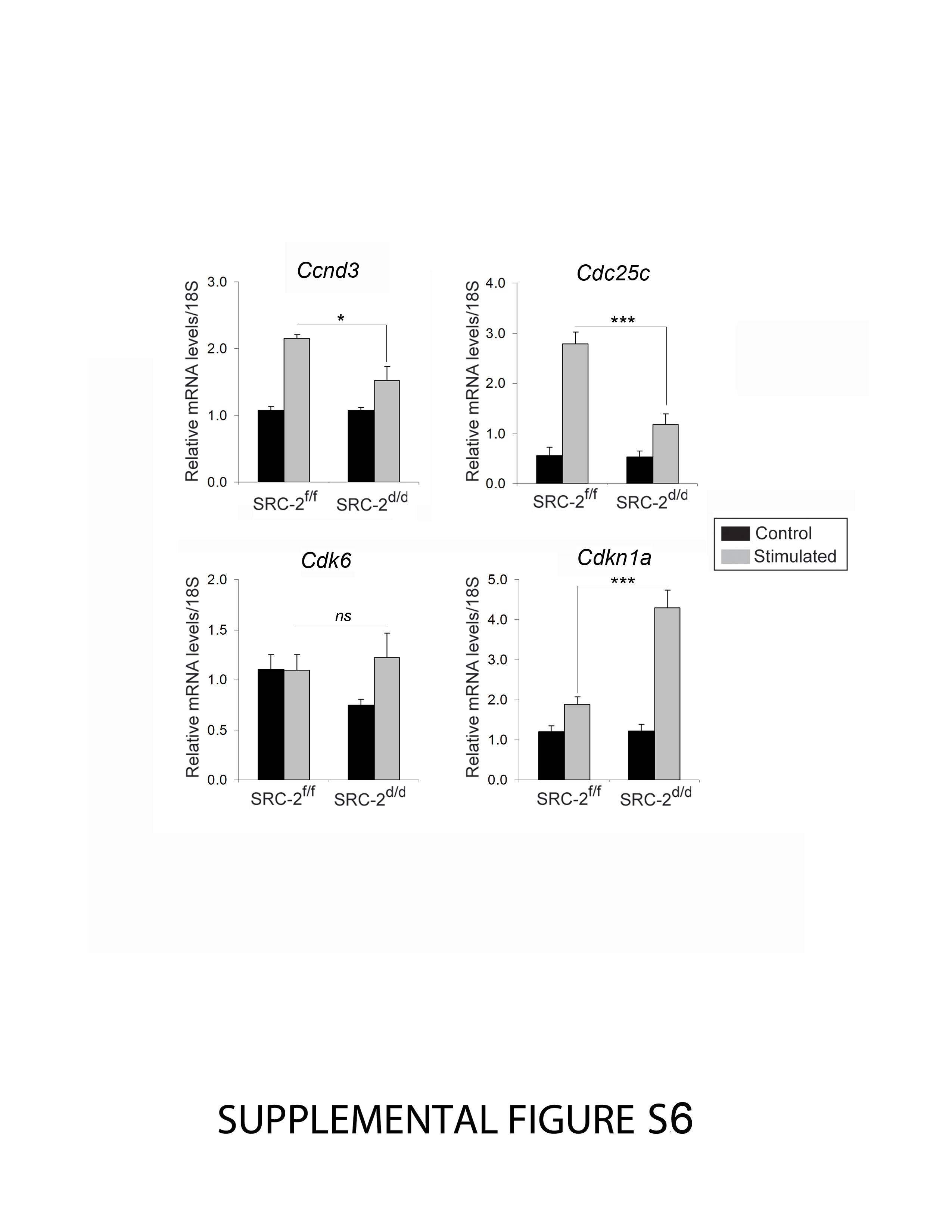

Supplement: Figure S6 — Quantitative real-time PCR analysis of transcript levels for Ccnd3, Cdk6, Cdc25c and Cdkn1a in the control and stimulated horns following two days of deciduogenic stimulus from SRC-2f/f and SRC-2d/d mice. Results represent means ±SE; n = 5 mice/group. *P<0.05; ***P<0.001. (TIF) [file pgen.1003900.s006.tif]

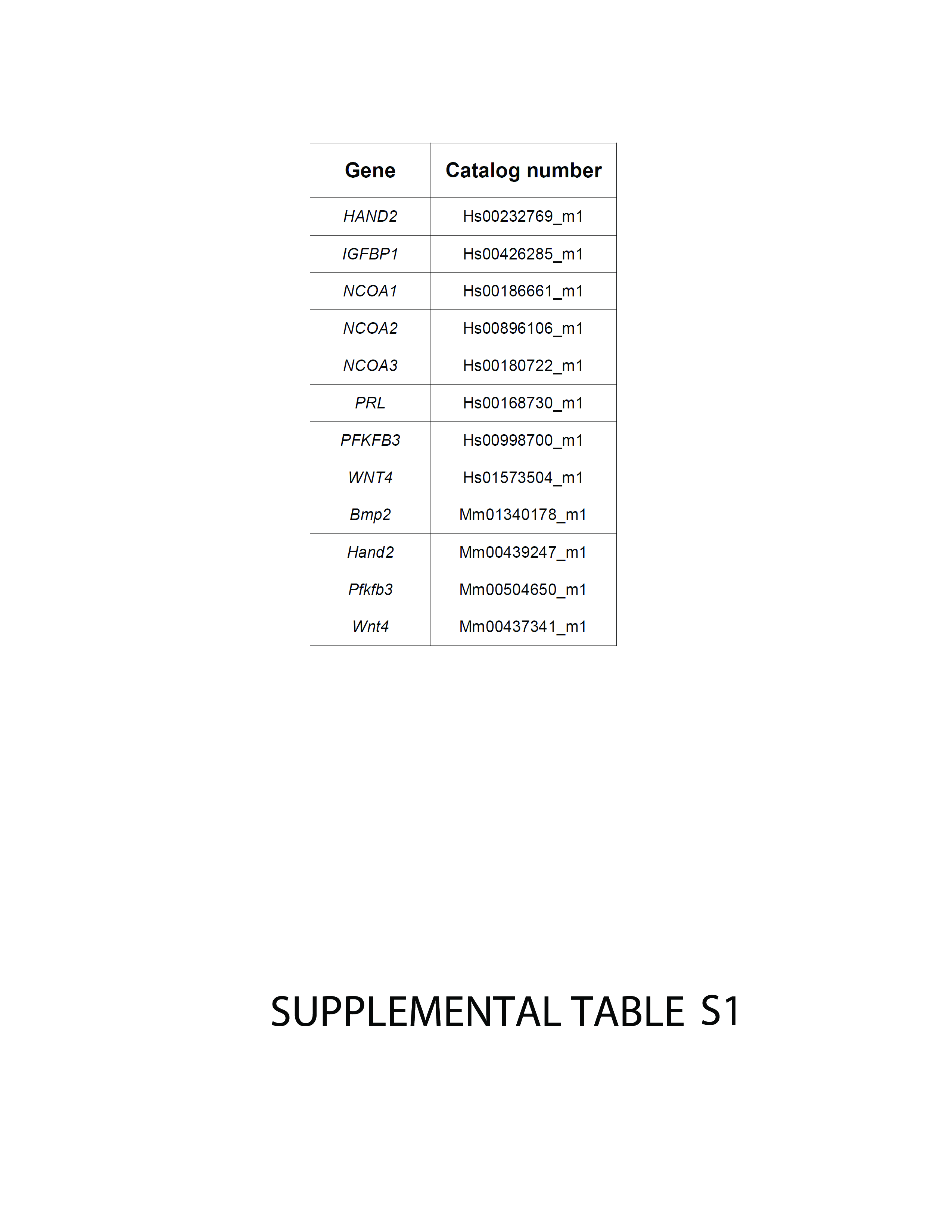

Supplement: Table S1 — List of gene specific primers used in Quantitative Real-Time PCR analysis. (TIF) [file pgen.1003900.s007.tif]
